# Dimensionality of Cognitions in Behavioral Addiction

**DOI:** 10.1007/s40473-016-0068-3

**Published:** 2016-02-20

**Authors:** L. S. Morris, V. Voon

**Affiliations:** Behavioural and Clinical Neuroscience Institute, University of Cambridge, Cambridge, UK; Department of Psychology, University of Cambridge, Cambridge, UK; Department of Psychiatry, University of Cambridge, Addenbrooke’s Hospital, Cambridge, UK

**Keywords:** Behavioral addiction, Gambling, Binge-eating, Gaming, Compulsivity, Impulsivity

## Abstract

Cognitive constructs provide conceptual frameworks for transpathological characterization and improved phenotyping of apparently disparate psychiatric groups. This dimensional approach can be applied to the examination of individuals with behavioral addictions, for example, towards gambling, video-games, the internet, food, and sex, allowing operationalization of core deficits. We use this approach to review constructs such as impulsivity, compulsivity, and attention regulation, which may be most relevant, applicable, and successful for the understanding and subsequent treatment of the addictions.

## Introduction

Recognition of non-substance-related addictions, for example, towards gambling, video-games, the internet, food, and sex, is rapidly growing due to expanding evidence of common impairments traditionally associated with substance use disorders (SUD) [1, 2]. The examination of cognition in behavioral addictions allows a trans-pathological characterization of deficits that cuts across diagnoses and phenotypes, providing a novel and accessible means of categorizing and treating apparently disparate groups.

A need to define and dissociate such disorders and psychopathological influences is key for an effective approach to the diagnosis and treatment of behavioral addictions. A potentially promising route to such characterization is via cognitive constructs, conceptual frameworks that transcend disorder categories for cross-diagnostic significance [3]. This approach, using constructs such as impulsivity, compulsivity, and attention regulation, may be most relevant, applicable, and successful for addictions [3–5]. The focus of the current paper is to review these cognitive constructs in behavioral addictions, with comparisons to SUD, in order to establish any similarities as well as any features in which they differ.

We conducted a search of Pubmed (http://www.ncbi.nlm.nih.gov/pmc/) with the following search terms: pathological gambling or pathological gaming or problem gaming, and cognition, working memory, learning, memory, planning. Compulsive sexual behaviors were searched separately, and binge-eating disorder is briefly reviewed.

## Classification

Pathological gambling (PG) was the first behavioral addiction included as a standalone disorder in the Diagnostic and Statistical Manual of Mental Disorders (DSM) in 1994. Early studies classed PG as either an impulse control disorder [6], obsessive-compulsive (OC) disorder, or non-pharmacological addictive disorder [7], but subsequent studies highlighted similarities with substance use disorders (SUD) [8] over OC spectrum disorders [9]. A meta-analysis demonstrated strong associations between PG and OC traits rather than OC disorder (OCD) [10], confirming its suggested categorization as a behavioral addiction [8]. Indeed, more recently, DSM-5 has included gambling disorder under substance-related and addictive disorders.

Internet- and gaming-related behaviors are increasingly recognized but were not included in the DSM-5 as further characterization was required [11]. However, while internet- and gaming-related addiction is less well recognized, their current impact is high. An epidemiological study of internet use in the USA showed that between 3.7 and 13 % of respondents met criteria for problematic internet use [12]. Pathological video game use appears more prevalent in younger populations, with almost triple the prevalence in adolescents [13], reaching 8–9.3 % of adolescents/young adults in the USA and Germany [14, 15]. Due to overlaps between internet addiction, internet gaming addiction, and video gaming addiction, these behaviors are discussed together in this review.

Compulsive behavior towards food and sex forms around a reward that naturally exists in the environment. Due to the observation that sex activates similar brain regions and neurotransmitter systems as drugs of abuse, sex addiction was thought be a disorder of dependence relatively early [16, 17] but that certain types, such as “cyber-sex” (which has overlaps with sex addiction [18]), fit the terms of addiction more appropriately [19]. The term compulsive sexual behavior (CSB) was coined in 1985 [20] and found to be a stable trait [21], distinguishable from healthy sexual activity that could be successfully modified with psychotherapy [20]. While there is no globally agreed upon definition of CSB [22], some diagnostic criteria have been outlined, including recurrent sexual cognitions and urges that lead to subjective distress or health, social, or economic costs [22–25]. Finally, compulsive behavior towards another natural reward, food, emerges in binge-eating disorder (BED), which is characterized by periods of rapid food intake without purging and is commonly but not always associated with obesity. Cognitive processes in BED have recently been reviewed [26] but key findings are included here.

## Classical Signs of Addiction

Physiological signs of addiction such as tolerance and withdrawal are important features of SUD, and the presentation of such phenomena in behavioral addictions would implicate shared underlying neuroadaptive or psychopathological processes. However, there remains to be little evidence demonstrating such features in behavioral addictions. Some convincing evidence comes from studies of PG, in particular of tolerance, withdrawal, craving, reduced control, and disruption of important (personal, familial, and/or vocational) activities [7]. Individuals with PG experience symptoms of withdrawal (including restlessness, headaches, and irritability) [27, 28], at comparable levels to individuals with alcohol use disorder (AUD) [27]. Also, 91 % of a sample of 222 PG who were slowing or stopping gambling habits reported “cravings” which were not related to comorbid alcohol or drug use [28]. Craving in PG [29] may be associated with depression [30], potentially suggesting an influence of negative reinforcement, a process that is often suggested to underlie substance addiction [31]. In terms of tolerance, individuals with PG demonstrate changes in heart rate responses to gambling activities [32] and report increasing levels of gambling or bet size over time [27]. This latter effect was linked with an aim of increasing chances of winning rather than increasing or maintaining excitement levels [27], suggesting that motivational orientation in this group may differ from SUD.

There is also some evidence of tolerance, withdrawal, and familial and social problems in adolescents who meet criteria for internet-related addiction compared to non-addicted peers [33]. Self-reported measures of tolerance and withdrawal associated with internet use in college-aged adults seem to be higher in those engaging in social functioning online [34]. However, more empirical evidence is certainly lacking in this group [35]. Implication of tolerance and withdrawal in BED remains largely anecdotal [36]; however, a recent study showed that approximately half of a sample of 81 obese BED patients met criteria for tolerance and withdrawal symptoms on the Yale food addiction scale [37], suggesting potential subgroups with varying severity.

Substance and non-substance dependence behaviors seem to have shared vulnerabilities [38], and maladaptive, inflexible behavior is characteristic of both [39, 40], with often significant disruption of personal endeavors [41] and financial or social loss [42]. Even with this growing evidence of resemblance between substance and non-substance-related addictions (at least for PG), further empirical and combined studies are still required, which together may highlight more severe subgroups and potentially novel treatment strategies.

## Cognition

Disturbances in cognitive function in behavioral addictions are not always apparently consistent. Individuals with PG have demonstrated impairments in cognitive flexibility and planning [43, 44] but there are also reports of no difference compared to HV in the same measures [45]. Furthermore, a direct comparison of PG and a SUD (alcohol dependence) showed impairments in working memory in SUD but not PG suggested to be related to alcohol exposure [46].

**Fig. 1:**
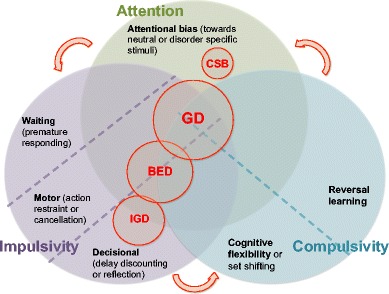
Schematic depiction of cognitive impairments in reviewed behavioral addictions. *Red circles* indicate specific, reported cognitive impairments for each behavioral addictions reviewed. For example, individuals with compulsive sexual behavior (CSB) show impairments in attentional bias, whereas individuals with pathological gambling or gambling disorder (GD) show attentional bias, motor and decisional impulsivity, and impaired cognitive flexibility and reversal learning. *BED* binge-eating disorder, *IGD* internet gaming disorder

This highlights the need to further sub-depict constitute component processes of complex cognition, perhaps by separating the effects of structural task components. In the following, we examine separately the roles of attentional biases, impulsivity, and compulsivity. These three constructs are depicted in Fig. 1, in which each behavioral addiction is positioned based on known impairments.

### Attentional Bias

SUD are often characterized by attentional biases towards drug cues, a disturbance that facilitates craving in a cyclic, self-propagatory manner [47]. The relationship between attentional bias and craving remains despite treatment status [48]. Disruption of attentional regulation also appears to be relevant across a range of behavioral addictions. Individuals with PG both self-report [49] and demonstrate impairments in higher order attention processes [50, 51]. Similar to SUD and pathological gaming use, this deficit may reflect a shift in attention, as individuals with PG show biased and maintained attention towards gambling cues [52]. There is also evidence of early attentional biases towards food cues in BED, as well as difficulty disengaging from food cues, although the latter effect is also observed in healthy individuals [53]. Attentional bias towards internet-related stimuli has been reported in individuals with internet gaming addiction [54], and CSB is associated with an early attentional bias towards sexually explicit images [55]. Thus, a common influencing factor of attentional bias to the disorder-specific object seemingly presents across these behavioral aberrancies.

While the relationship between attentional bias and craving is yet to be explored across behavioral addictions, insights from studies of SUD suggest a strong link between the two, implicating a pathway towards pathological behavior, particularly driven by incentive motivation. It is difficult from these studies however to determine whether attentional biases pre-existed the disorder or were facilitated by them, although evidence from the SUD literature suggests the latter [47]. In line with this, modification of attentional biases to divert attentional resources from drug stimuli has had promising and clinically relevant effects [56, 57], with some effect on craving in smokers [58], although the generalizability of attentional training is currently unclear [59].

### Impulsivity

Impulsivity, a tendency towards rapid, unplanned behaviors that are divorced from sufficient forethought and occur despite potential negative consequence, is well documented across a range of psychiatric disorders, including SUD [60, 61]. Impulsive behavior is now also demonstrated in behavioral addictions, including different groups of individuals with PG [43, 45, 62], problem gambling [63], and BED [26]. Self-reported impulsivity acts as a risk factor for pathological gaming in elementary and secondary schools in Singapore [64] and is associated with gambling severity in PG captured by the PG-YBOCS [65]. Self-reported impulsivity may also be higher in PG compared to that in SUD [66].

Impulsivity can be further decomposed into several discrete yet often overlapping constructs, subserved by dissociable neural systems [61]. Briefly, motor impulsivity describes a capacity for response inhibition or action cancelation; decisional impulsivity describes impulsive choice, modulated either by the influence, or lack, of prior evidence (reflection impulsivity) or by the temporal features of an outcome (delay discounting); and finally, waiting impulsivity describes the propensity towards disadvantageous premature responding. This heterogeneity suggests that there may be differing presentations across disorders [61].

Motor impulsivity can be captured with a Go/NoGo task or stop signal task (SST), in which responses are inhibited before or after response initiation, marking action restraint or action cancelation, respectively. Individuals with SUD show impaired performance on both the SST and Go/NoGo tasks, highlighted by a meta-analysis showing deficits particularly for stimulants and nicotine but not opioid or cannabis abuse [67••, 68]. Studies of motor impulsivity in PG have shown mixed results. Impaired performance during the Go/NoGo task have been reported [69] as well as no difference from healthy controls on the same task [67••]. Similarly, several studies have reported a lack of any difference during the SST compared to healthy controls [70–72] although a recent meta-analysis found a medium-large effect of impaired performance on the SST in gamblers [67••]. Since this group shows difficulties with target detection during the Go/NoGo task [69] and Go reaction time during the SST [67••], some of these effects may be related to inattention [67••]. Pathological video gamers have been reported to be unimpaired on the SST [73]. Interestingly, whereas problem gamers seemingly have reduced inhibitory control on the Go/NoGo task [63], pathological internet users have been shown to be more accurate on the same task compared to HV [74]. Thus, care must be taken around the generalizability of over-practiced computerized routine to laboratory tests, an effect that warrants further investigation. The role of motivational relevance of the stop cue is also of high importance; individuals with BED show consistent deficits in the Go/NoGo and SST but only in the context of a food cue, not with a neutral stimulus [75, 76]. Thus, impairments in response inhibition do not appear to be a uniformly observed across behavioral addictions, and some observed differences may indeed be related to extra-motor faculties.

In PG, BED and pathological gaming, emerging evidence suggests that deficits in impulsivity lie prominently within the realms of decision-making. Individuals with PG [49, 77–79], BED [80, 81], and pathological gaming [73] discount delayed rewards to a greater extent than healthy controls, meaning that smaller, immediate rewards are preferred over larger, delayed rewards. Although the same effect has been demonstrated in individuals with addictive disorders to drugs of abuse [68, 82, 83], this impairment may be more pronounced in individuals with PG. For example, individuals with PG show elevated delay discounting compared to cocaine dependent individuals [84], and gambling severity is a stronger predictor of discounting rate than substance use history or another self-reported measure of impulsivity [85]. Delay discounting is similarly observed in obese subjects with and without BED, although in obese subjects with high body mass index, those with BED show greater discounting across monetary, food reward, and massage time [81], implicating impairments in decisional impulsivity across reward types [86]. It should be noted that individuals with PG display difficulties in accurately perceiving time [43], a factor that may certainly contribute to decisions concerning delayed outcomes but yet to be directly tested.

Further evidence of impairments in decisional impulsivity arise from the use of the information sampling task (IST), which measures the tendency to sample or gather information before making a decision [46]. Individuals with PG and AUD show impairments in this measure of reflection impulsivity [46], highlighting shared deficits across substance-related and behavioral addictions. Pathological gamers similarly show less evidence accumulation before decision in the IST [73] and less beads drawn before decision in the beads task [87].

There are few studies assessing waiting impulsivity in humans with behavioral addictions. However, a recent report using a novel translational task assessing premature responses found no differences between individuals with BED and obese controls while stimulant-, alcohol-, and nicotine-dependent individuals were impaired [88]. Pathological video gamers made more premature responses compared to healthy controls but only in the context of comorbid nicotine use [73]. While waiting impulsivity has been shown to be impaired across a range of SUD, the effects may be drug state-dependent, as ex-smokers show normal levels of premature responses [88]. Further studies in PG and CSB are necessary before the extent of the dimensionality of waiting impulsivity across disorders of addiction is properly understood.

There is currently little empirical evidence of deficits in impulsivity in individuals with CSB [22]. Using a semi-structured clinical interview, traits of impulsivity were found to be common in a sample of 23 men and 2 females who met criteria for CSB [89], and a more recent study showed that CSB self-reports higher levels of impulsivity [55].

### Cognitive Flexibility and Compulsivity

Measures of cognitive flexibility can highlight integrity of executive functions and the potential contribution of compulsive choice to pathological behavior. Cognitive flexibility has been assessed with the Wisconsin card sorting task (WCST) and the intra-dimensional extra-dimensional (IDED) set-shifting task. The WCST uses changing rules requiring flexible shifting of choices in the face of positive or negative feedback, in which the primary measure is of perseverative errors (continuing use of the same rule despite negative feedback, indicating compulsivity) or difficulty in task switching and inhibitory control. The IDED shifting task probes attentional set maintenance and conceptual set shifting, indicating cognitive shifting or flexibility.

Performance on both the WCST and IDED in SUD is inconsistent. Cocaine-dependent individuals are perseverative on the WCST but only during the initial stage of set-shifting [90]; however, poly-substance abusers have been shown to be no different from healthy controls [91]. There is no clear impairment on the WCST in alcohol dependence [92, 93] but acute alcohol does enhance preservative errors in healthy individuals [94]. Amphetamine but not opioid users show impairments at the crucial ED shift stage of the IDED task [95], although this effect was not replicated in a more recent study [96].

There is similar inconsistency in groups of behavioral addictions. While limited, evidence does however implicate impairments in set-shifting in BED [97•], as well as higher perseveration errors on the WCST compared to non-BED obese individuals [98] and problems maintaining set compared to obese controls [98] and individuals with anorexia nervosa [99]. However, the WCST has yielded inconsistent results amongst individuals with PG. Both enhanced errors of perseveration in PG females [100] and lack of a difference from healthy controls [101, 102] have been demonstrated. Increases in non-perseverative errors have been reported in PG during the WCST, suggesting that the observed impairments may not be specific to set-shifting but more of a broader cognitive dysfunction. Individuals with PG do seem to be impaired on IDED task [103], which improves with pharmacological intervention (memantine) [104].

While the literature for performance on tasks of cognitive flexibility in individuals with internet or gaming addiction is sparse, there is some evidence of impairments in set-shifting when a shift must be made between neutral and game-related stimuli [54] suggesting a specific effect of motivation rather than a general deficit in set shifting. Indeed, a recent study found no difference between individuals with internet addiction and healthy individuals during the IDED task [105•].

Another test of flexible behavior or compulsive choice is the probabilistic reversal learning (PRR) task, in which choice updating depends on a change or reversal of learned stimulus-outcome contingencies. Cocaine-dependent individuals are impaired at reversing in the face of a previously rewarded stimulus, largely perseverating for reward [106]. However, amphetamine, opiate [106], and nicotine [107] dependence has not been associated with this impairment. Reversal impairments have however been demonstrated in PG for both reward [62, 102, 107] and loss [107] outcomes. Because PG demonstrates largely normal performance on the WCST [102], this may be due to either intrinsic differences in set shifting versus reversal learning (e.g., utilizing dorsolateral versus orbitofrontal substrates respectively) or the difference in motivational outcomes between the two tasks: the PRR uses monetary outcomes but the WCST does not [102]. Indeed, a recent meta review found an association between PG and perseveration on monetary tasks despite intact executive planning [108]. This presents an interesting distinction for the study of PG. Unlike for other addictions, cognitive tasks routinely employed in research often use the very object of addiction for PG: monetary rewards. If the PRR used cocaine cues or rewards for cocaine dependence or food rewards for BED, impairments in reversing may be more prevalent.

Perseveration during the PRR in problem gamblers is associated with reduced sensitivity to both reward and loss [107], and perseveration for reward in particular has also been demonstrated with the card playing task, wherein choices that were previously rewarded must be inhibited; PG continue playing longer despite a shift from rewarding outcomes to losses [109]. Thus, monetary reward seems to be influential in PG, and impairments of cognitive flexibility must be considered in terms of reward sensitivity particularly in this group.

While there are very few studies examining compulsivity using these tasks in CSB, evidence from semi-structured interviews also suggests compulsive traits in this group [89] but further studies are still required. Mirroring inconsistencies in SUD, it seems that these particular measures of compulsivity or flexible choice may not pick up a consistent or robust impairment in behavioral addictions, although impairments in general cognitive flexibility in BED and reward perseveration in PG are implied.

## Comorbidity and Heterogeneity

Important for the development of clear characterizations of all addictions, PG currently acts as an appropriate, toxicity-free model for addiction [110]. However, for PG [7, 111], and also CSB [25], comorbidity with SUD is high. SUD share high genetic overlap with PG [111], with the risk of alcohol dependence accounting for 12–20 % of the genetic variation in the risk of PG, highlighting underlying common factors [112, 113]. Furthermore, at-risk or problem gambling in a large sample of adolescents was more frequent in self-reported marijuana users and associated with more severe gambling [114].

While much evidence implicates aberrancies in decision-making and choice preference in the face of an immediate monetary reward in PG, these and other implicated deficits must be assessed in light of known heterogeneities of the population. Firstly, gender seems to play a role in the motivations towards and subsequent harms of gambling in problem gamblers [115]. The disorder appears more common in males, who also report higher rates of drug misuse [115, 116], compared to females who display higher prevalence of mood, anxiety, and affective disorders [116, 117] and a later age of disorder onset [117]. Such influences may affect not only reasons for disorder onset but distinct routes of effective treatment and symptom management. For example, females are more likely to report relief of a negative state or mood as a reason for pathological gambling behaviors [117].

Other comorbidity in PG such as post-traumatic stress disorders or obesity may also contribute to problems with risky decision-making [118] and impulsivity [119••], respectively. In problem gamblers, high rates of ADHD (21.4 % of 126) are also associated with higher reported impulsivity and response inhibition (SST) [120], and the prevalence of obesity in this group (10.6 % of 207) may explain reaction time differences that contribute to differences in motor impulsivity [119••]. The age of the individual and the age of disorder onset also contribute to differences in disorder presentation. A higher prevalence of PG during adolescence may reflect slower development of cognitive control mechanisms, particularly for management of trait impulsivity [121]. Older gamblers are less likely to report anxiety, family problems, and illegal behaviors [122]. Furthermore, race and education significantly predict gambling severity [123], and differences within race groups are present, with white (Australian) compared to Chinese gamblers reporting higher perceived stress, expectancy bias, and negative affect [124]. Thus, demographically separable subgroups may form once more thorough characterizations have been established, yielding more individualized prospects for treatment strategies.

Another important factor in understanding the patterns of cognitive deficit, particularly in PG, is the type of game that pathological behaviors form towards. Game preference in PG (slot machines versus casino) differentiate deficits [125, 126], with seemingly poorer decision-making and motor impulsivity in pathological slot machine gamblers compared to pathological casino gamblers [109]. Slot machine gambling is a form of non-strategic gambling, which differs in style from strategic gambling (for example, card games, dice games, and sports betting) [126]. When directly comparing these two groups, slot machine users make more commission errors on the Go/NoGo task of response inhibition [109]. It seems that the non-strategic sub-group is more impaired at general tests of executive function [45] and may be driving some discussed deficits.

## Conclusion

In review of disordered cognition across addictions, we demonstrate a transpathological and dimensional approach to the understanding of seemingly disparate groups. Discussed evidence is collated in Table 1, which illustrates that disturbed attentional biases and decisional impulsivity for delayed rewards present across the behavioral addictions currently reviewed. The influence of motivational relevance is clear, with impairments often forming around the disorder-specific object (i.e., food in BED). Whether the relationship between cognitive constructs, for example, attentional bias and craving, is a cause or effect of pathological addictive behaviors is the question that is yet to be clarified. Together, cognitive constructs provide a useful framework for phenotypic characterization of emerging psychiatric groups.

**Table 1:** Cognitive disturbances across behavioral addictions

	Gambling disorder	Internet gaming disorder/internet addiction	Binge-eating disorder	Compulsive sexual behavior
Attention				
Attentional Bias	X (gambling cues)	X (gaming/internet cues)	X (food images)	X (sexually explicit images)
Impulsivity				
Motor (action cancelation, SST)	Conflicting	o	X (food cue only)	
Motor (action restraint, Go/NoGo)	Conflicting	X/better	X (food cue only)	
Decisional (delay discounting)	X	X	X	
Decisional (reflection impulsivity)	X	X		
Waiting (premature responding)		o	o	
Compulsivity				
Cognitive flexibility (WCST)	Conflicting		X	
Set shifting (IDED)	X	/ o		
Reversal learning	X			

*SST* stop signal task, *WCST* Wisconsin card sorting task, *IDED* intra-dimensional/extra-dimensional set shifting task. *X* = impaired, *o* = typical performance
